# Cytokine signaling-1 suppressor is inducible by IL-1beta and inhibits the catabolic effects of IL-1beta in chondrocytes: its implication in the paradoxical joint-protective role of IL-1beta

**DOI:** 10.1186/ar4381

**Published:** 2013-11-18

**Authors:** Yong Seok Choi, Jin Kyun Park, Eun Ha Kang, Young-Kyun Lee, Tae Kyun Kim, Jin-Haeng Chung, Jason M Zimmerer, William E Carson, Yeong Wook Song, Yun Jong Lee

**Affiliations:** 1 Department of Internal Medicine, Seoul National University Bundang Hospital, 166 Gumi-ro, Bundang-gu, Seongnam-si, Gyeonggi-do, Korea; 2 Department of Internal Medicine, Seoul National University Hospital, Seoul, Korea; 3 WCU Department of Molecular Medicine and Biopharmaceutical Sciences, Medical Research Institute, Seoul National University College of Medicine, Seoul, Korea; 4 Department of Orthopedic Surgery, Seoul National University Bundang Hospital, Seongnam-si, Korea; 5 Department of Pathology, Seoul National University Bundang Hospital, Seongnam-si, Korea; 6 Department of Surgery Arthur G. James Cancer Hospital and Richard J. Solove Research Institute, The Ohio State University, Columbus, OH 43210, USA; 7 Department of Internal Medicine, Seoul National University College of Medicine, Seoul, Korea

## Abstract

**Introduction:**

Although IL-1β is believed to be crucial in the pathogenesis of osteoarthritis (OA), the IL-1β blockade brings no therapeutic benefit in human OA and results in OA aggravation in several animal models. We explored the role of a cytokine signaling 1 (SOCS1) suppressor as a regulatory modulator of IL-1β signaling in chondrocytes.

**Methods:**

Cartilage samples were obtained from patients with knee OA and those without OA who underwent surgery for femur-neck fracture. SOCS1 expression in cartilage was assessed with immunohistochemistry. IL-1β-induced SOCS1 expression in chondrocytes was analyzed with quantitative polymerase chain reaction and immunoblot. The effect of SOCS1 on IL-1β signaling pathways and the synthesis of matrix metalloproteinases (MMPs) and aggrecanase-1 was investigated in SOCS1-overexpressing or -knockdown chondrocytes.

**Results:**

SOCS1 expression was significantly increased in OA cartilage, especially in areas of severe damage (*P* < 0.01). IL-1β stimulated SOCS1 mRNA expression in a dose-dependent pattern (*P* < 0.01). The IL-1β-induced production of MMP-1, MMP-3, MMP-13, and ADAMTS-4 (aggrecanase-1, a disintegrin and metalloproteinase with thrombospondin motifs 4) was affected by SOCS1 overexpression or knockdown in both SW1353 cells and primary human articular chondrocytes (all *P* values < 0.05). The inhibitory effects of SOCS1 were mediated by blocking p38, c-Jun N-terminal kinase (JNK), and nuclear factor κB (NF-κB) activation, and by downregulating transforming growth factor-β-activated kinase 1 (TAK1) expression.

**Conclusions:**

Our results show that SOCS1 is induced by IL1-β in OA chondrocytes and suppresses the IL-1β-induced synthesis of matrix-degrading enzymes by inhibiting IL-1β signaling at multiple levels. It suggests that the IL-1β-inducible SOCS1 acts as a negative regulator of the IL-1β response in OA cartilage.

## Introduction

Osteoarthritis (OA) is the most common arthritis, characterized by progressive loss of articular cartilage, subchondral bone remodeling, and synovial inflammation, leading to debilitating joint pain and functional limitation [1,2]. The underlying pathophysiologic process of cartilage destruction in OA has not been completely elucidated. Inflammation is believed to be implicated in the OA pathogenesis, even in early stages, by shifting the balance from the anabolic toward the catabolic state with gradually progressive cartilage loss. In OA, chondrocytes, the only cells residing in cartilage, are a target of catabolic cytokines, including interleukin (IL)-1β, tumor necrosis factor (TNF)-α, and IL-6. IL-1β in particular has been considered a key amplifier and perpetuator of cartilage damage because it suppresses matrix protein synthesis and induces matrix-degrading enzymes and other proinflammatory cytokines, including IL-6 [3,4]. However, postsurgical or spontaneous OA development is paradoxically accelerated in IL-1β or IL-6 knockout mice [5-7], suggestive of their intricate role in cartilage biology; the proinflammatory cytokines might slow the OA progression via yet-unknown mechanisms.

Suppressors of cytokine signaling (SOCS) belong to a protein family that is composed of eight SH2-containing proteins and forms E3 ubiquitin ligase complexes to degrade target proteins by proteasomes. As negative feedback, these proteins are induced by a variety of cytokines and inhibit, in turn, intracellular signaling of diverse cytokines and growth factors [8,9]. SOCS1 and SOCS3 are the best characterized, and SOCS1 is considered more potent than SOCS3 [10,11]. Although IL-1β is not a main inducer of SOCS-family proteins or a potent activator of signal transducer and activator of transcription (STAT), IL-1β has been reported to induce SOCS1 or SOCS3 in several types of cells including chondrocytes [12-14]. Furthermore, SOCS1 may inhibit IL-1β-signaling pathways; SOCS1^null^ T cells were found to be hypersensitive to IL-1β [15]. When HEK293 cells transfected with SOCS1 were stimulated with IL-1β, SOCS1 bound to NF-κB p65 and regulated NF-κB signaling in the nucleus [16]. However, the mechanisms of SOCS1-mediated inhibition of IL-1β signaling pathways have not been fully studied.

Here, we demonstrated that the SOCS1 is present in OA cartilage, especially in the area of severe cartilage damage, and is inducible by IL-1β in primary human articular chondrocytes (HACs). Furthermore, SOCS1 suppresses the production of proteolytic matrix metalloproteinases (MMPs) and aggrecanase-1 (ADAMTS-4) in human SW1353 chondrocytic cell lines and HACs by inhibiting c-Jun N-terminal kinase (JNK) and p38 mitogen-activated protein (MAP) kinases activation, by preventing the degradation of the inhibitor of NF-κB (IκBα), and by accelerating degradation of TGF-β-activated protein kinase 1 (TAK1).

## Methods

### Plasmids and reagents

A PINCO retroviral vector expressing myc-tagged human SOCS1 was kindly provided by William E. Carson (Ohio State University, Columbus, OH, USA). pShuttle2 and pBABE retroviral vectors were purchased from Addgene (Cambridge, MA, USA). SOCS1 small-hairpin (sh) RNA and copGFP Control Lentivirus particles came from Santa Cruz Biotechnology (Santa Cruz, CA, USA). The Platinum-A retroviral packing cell line was obtained from Cell BioLabs (San Diego, CA, USA). NF-κB-mediated luciferase activity was assayed by using pGL luc-based 3X-κB-L plasmid [17]. Recombinant IL-1β was purchased from Peprotech (Rocky Hill, NJ, USA). ELISA kits for MMP-1, MMP-3, MMP-13, and TIMP-1 were obtained from R&D Systems (Minneapolis, MN, USA). Anti-SOCS1 was purchased from LifeSpan Bioscience (Seattle, WA, USA) for immunohistochemistry, (IHC) and Chemicon International (Temecula, CA, USA), for immunoblot. Anti-TAK1 was purchased from Novus Biologicals (Littleton, CO, USA) for immunoprecipitation (IP) and from Santa Cruz for immunoblot. Anti-phospho-NF-κB p65 (Ser311 or Ser536) and anti-myc were obtained from ABcam (Cambridge, MA, USA), and anti-IκBα was from Santa Cruz. Anti-ADAMTS4 was from Calbiochem (San Diego, CA, USA). The other antibodies were purchased from Cell Signaling Technology (Beverly, MA, USA). An ERK inhibitor U0126 was obtained from Promega (Madison, WI, USA), and JNK inhibitor SP600125 was from BioMol International (Plymouth Meeting, PA, USA). A p38 MAP kinase inhibitor SB202190 and NF-κB inhibitor SN50 were purchased from Alexis Biochemicals (Farmingdale, MI, USA). MG132 was from Sigma-Aldrich (St. Louis, MO, USA). SW1353 chondrosarcoma cell line (ATCC HTB-94) was obtained from American Type Culture Collection (Manassas, VA, USA).

### Patients and cartilage samples

OA cartilage was obtained from 14 patients with primary knee OA who underwent total knee-replacement arthroplasty. Control healthy cartilage specimens were obtained from four patients with femur-neck fractures who had no history of hip OA. A written informed consent was obtained from all study participants. This study was approved by the Institutional Review Board of Seoul National University Bundang Hospital (IRB No. B-0607/035-018).

### Culture of primary HACs

HACs from OA cartilage portions with less than 50% of thickness loss were released by enzymatic digestion, as previously described [18]. Isolated chondrocytes were plated in 100-mm-diameter dishes and cultured to 70% confluence in Dulbecco Modified Eagle Medium (DMEM) containing 10% fetal bovine serum (FBS), 100 IU/ml penicillin, and 100 μg/ml streptomycin at 37°C in a humidified 5% CO_2_ atmosphere. After HACs were transferred to six-well plates, they were stimulated for 4 hours with IL-1β (0 to 10 ng/ml) in serum-free media. The SOCS1-overexpressing HACs were cultured in pellets 24 hours before the stimulation with IL-1β.

### Overexpression and knockdown of human SOCS1

To generate the pBABE viral vector containing the *myc*-tagged human SOCS1, SOCS1 cDNA was amplified with two primer sets (forward 5′-CTAGGATCCATGGTAGCACACAACCAGGTG-3′ and reverse 5′-GCCGAATTCTCAAATCTGGAAGGGGAAGGAG-3′) that contained a *Bam*H1 or *Eco*RI restriction-enzyme site. PCR products were digested with *Bam*H1 and *Eco*RI and cloned into the pBABE viral vectors. To produce retrovirus, the pBABE-SOCS1 viral vectors were transfected into a Platinum-A retroviral packing cell line. Supernatants were collected 72 hours after transfection. To infect SW1353 cells, viral supernatant was mixed with fresh medium with 8 μg/ml of polybrene at 1:1 ratio, and the mixture was applied to freshly seeded cells.

To deliver SOCS1 or control shRNA into the SW1353 cells, SOCS1 shRNA or copGFP lentiviral particles were mixed with fresh medium and 5 μg/ml of polybrene, and the mixture was applied to freshly seeded cells. Stable overexpressing or knockdown cell lines were selected with puromycin (5 μg/ml). To establish SOCS1-overexpressing HACs, pShuttle2-SOCS1 or empty vector was electrotransfected by using a Gene Pulser Xcell System (Bio-Rad, Hercules, CA, USA) under the condition of 50-V and 2-ms pulse (4-mm cuvette).

### Measurement of MMPs and TIMP-1 in culture supernatants

Nontransfected and transfected SW1353 cells were plated onto 48-well plates (1 × 10^4^ cells/well) for 24 hours and then pretreated with serum-free media for 2 hours. The cells were stimulated with IL-1β (1 to 10 ng/ml) for 24 hours. The concentrations of MMP-1, -3, and -13 and TIMP-1 in the conditioned media were measured with commercial ELISA kits according to the manufacturer’s instructions.

### Reverse transcriptase-polymerase chain reaction for SOCS-1

Expression of SOCS1 was semiquantitatively determined by using RT-PCR with specific primer pairs: SOCS1 forward primer 5′-CACGCACTTCCGCACATTCC-3′ and reverse primer 5′-TCCAGCAGCTCGAAGAGGCA-3′ (GeneBank accession number NM_003745). β-actin was used as the internal RT-PCR control by using forward primer 5′-ACACTGTGCCCATCTACGAG-3′ and reverse primer 5′-TACAGGTCTTTGCGGATGTC-3′ (NM_001101).

Quantitative real-time RT-PCR was performed by using an ABI-7500 real-time PCR machine (Applied Biosystems, Foster City, CA, USA). Specific Taqman primers and probes for SOCS1 (assay ID Hs00705164_S1), MMP-1 (Hs00233958_m1), MMP-3 (Hs00968308_m1), MMP-13 (Hs00233992_m1), ADAMTS4 (Hs00192708_m1), glyceraldehyde-3-phosphate dehydrogenase (GAPDH; Hs99999905_m1), and large ribosomal protein (RPLPO, Hs99999902_m1) were purchased from Applied Biosystems. The number-fold difference in the expression of target mRNA was calculated with a comparative Ct method (2^-ΔΔCt^), normalized to GAPDH.

### Western blotting and immunoprecipitation (IP)

To prepare the total cell lysates, SW1353 cells were washed twice with ice-cold PBS, harvested, and lysed in IP buffer (25 m*M* HEPES (pH 7.7), 150 m*M* NaCl, 1% Triton X-100, 25 m*M* β-glycerophosphate, phosphatase inhibitor cocktail, and protease inhibitor cocktail) for 60 minutes on ice. For immunoprecipitation, TAK1 antibody (2 μg) was added to the whole-cell extracts (500 μg) and incubated on a rotator overnight at 4°C. Then the protein G-agarose beads were further incubated for 3 hours at 4°C. The mixtures were centrifuged at 2,095 *g* for 3 minutes. The precipitates were washed 3 times by using pre-cold IP buffer, and the beads were resuspended in 2× SDS sample buffer.

The immunoprecipitates or the whole-cell lysates were separated on 10% denaturing polyacrylamide gels and transferred to polyvinylidene difluoride membranes. The membranes were probed with appropriate primary antibodies and IgG horseradish peroxidase-conjugated antibodies. Signals were developed by using an enhanced chemiluminescence system (Amersham Biosciences, Little Chalfont, UK).

### Immunohistochemistry of SOCS1 in OA and normal cartilage

The cartilage samples were fixed in 4% buffered paraformaldehyde for 2 days and then decalcified with buffered EDTA (20% EDTA, pH 7.4). After dehydration and embedding in paraffin, sections were cut to a thickness of 4 μm, deparaffinized, and rehydrated. Tissue sections from each patient were stained with rabbit antibodies against SOCS1 (cat. no. LS-C31894). The subsequent steps were performed automatically at 37°C by using Benchmark XT Slide Staining System Specifications (Ventana Medical Systems, Tucson, AZ, USA). After antigen retrieval and endogenous peroxidase blocking, the sections were incubated with anti-SOCS1 at a dilution of 1:100 for 60 minutes at room temperature. To visualize the immunostaining, Ultravision LP kit (Lab Vision, Fremont, CA, USA) was used. The slides were stained by using a diaminobenzidine detection kit and counterstained with hematoxylin.

Specimens were evaluated under light microscopy by a pathologist (J-H C). Percentages of SOCS1-positive chondrocytes (more than weak staining) were scored in the cartilage area of mild (grade 0 to 2) and severe (grade 3 to 4) damage, according to the histopathology-grade system of OsteoArthritis Research Society International (OARSI) [19]. The number of total cells was counted in at least three randomly selected high-power fields (150 cells or more). The negative controls were treated by using the same method without the primary antibody.

### Statistical analysis

All experiments were independently repeated at least 3 times, and data were expressed as mean ± SEM. For comparison of continuous variables, the Mann–Whitney test, Kruskal-Wallis test, or Wilcoxon signed-rank test was used as appropriate. For multiple comparisons, Bonferroni correction was applied. Statistical analyses were performed by using PASW Statistics version 18 software (SPSS Inc., Chicago, IL, USA) and *P* or corrected *P* < 0.05 was considered significant.

## Results

### Increased SOCS1 expression in OA cartilage

**Figure 1:**
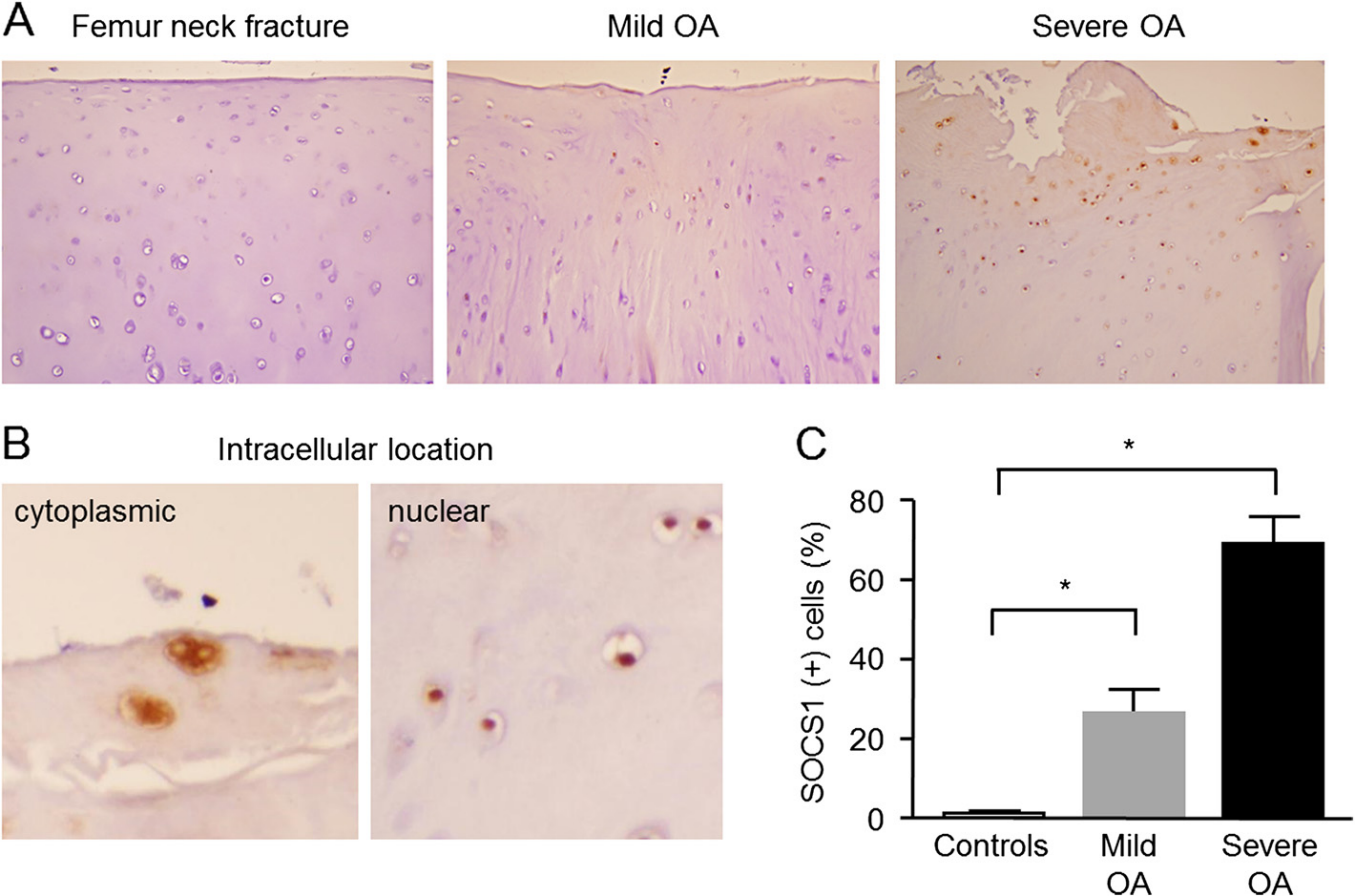
**Expression of SOCS1 in osteoarthritic cartilage.** Cartilage samples from four patients with femoral neck fracture and six with knee OA were immune-stained, as described in Methods. No to minimal SOCS1 expression was observed in healthy cartilage, whereas the intensity of SOCS1 expression and the number of SOCS1-positive cells increased in OA cartilage, especially in areas of severe cartilage damage (**A**, original magnification × 200). SOCS1 was localized in the cytoplasm and nucleus (**B**, original magnification × 200). The percentage of SOCS1-expressing chondrocytes was significantly higher in the severe OA lesions as compared with mild OA and healthy cartilage (**C**; *P* < 0.01). Data were expressed as the means ± SEM. *Corrected *P* < 0.05 by Mann–Whitney test with Bonferroni correction.

IHC staining showed that SOCS1-positive chondrocytes were observed mainly in the superficial layers of OA cartilage and that SOCS1 was present in the cytoplasm and/or nucleus of chondrocytes (Figure 1), consistent with previous studies [20]. The expression of SOCS1 was significantly increased in OA cartilage compared with healthy cartilage (*P* < 0.01 by the Kruskal-Wallis test; Figure 1C). In healthy cartilages of the femoral head (*n* = 4), 1.4 ± 0.5% of chondrocytes expressed SOCS1 as compared with 26.4% ± 6.1% in mild (OARSI grade 0 to 2; *n* = 5) and 70.0 ± 6.7% in severe OA cartilage lesions (OARSI grade 3 to 4, *n* = 6) (both *P*_c_ < 0.05 by Mann–Whitney test with the Bonferroni correction).

### IL-1β-induced SOCS1 expression in primary HACs

**Figure 2:**
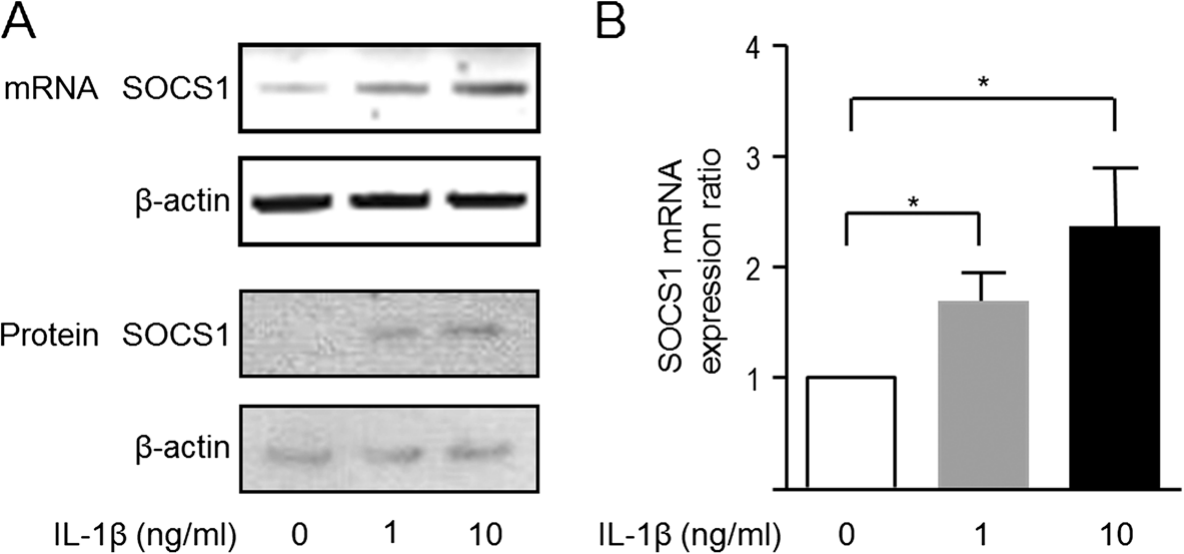
**IL-1β-induced SOCS1 expression in primary human articular chondrocytes (HACs).** HACs isolated from OA cartilage were activated with IL-1β, and the level of SOCS1 expression was checked with RT-PCR. IL-1β increased the expression of SOCS1 mRNA after 4 hours at mRNA and protein levels **(A)**. Quantitative real-time PCR analysis demonstrated that SOCS1 mRNA levels significantly increased with IL-1β stimulation in a dose-dependent manner (**B**; *n* = 4; *P* < 0.01). Data were expressed as means ± SEM. *Corrected *P* < 0.05 by Mann–Whitney test with Bonferroni correction.

Next, we examined whether IL-1β could induce SOCS1 expression in HACs. At baseline, the isolated chondrocytes (passage 1) expressed SOCS1 mRNA at a lower level (Figure 2A). After stimulation with IL-1β for 4 hours, the SOCS1 mRNA level increased significantly in a dose-dependent manner (*n* = 4; *P* < 0.01) (Figure 2B). Accordingly, SOCS1 protein expression was increased after IL-1β stimulation (Figure 2A).

### MMP-1, MMP-3, MMP-13, and ADAMTS-4 production in SOCS1-overexpressing or -knockdown chondrocytes

**Figure 3:**
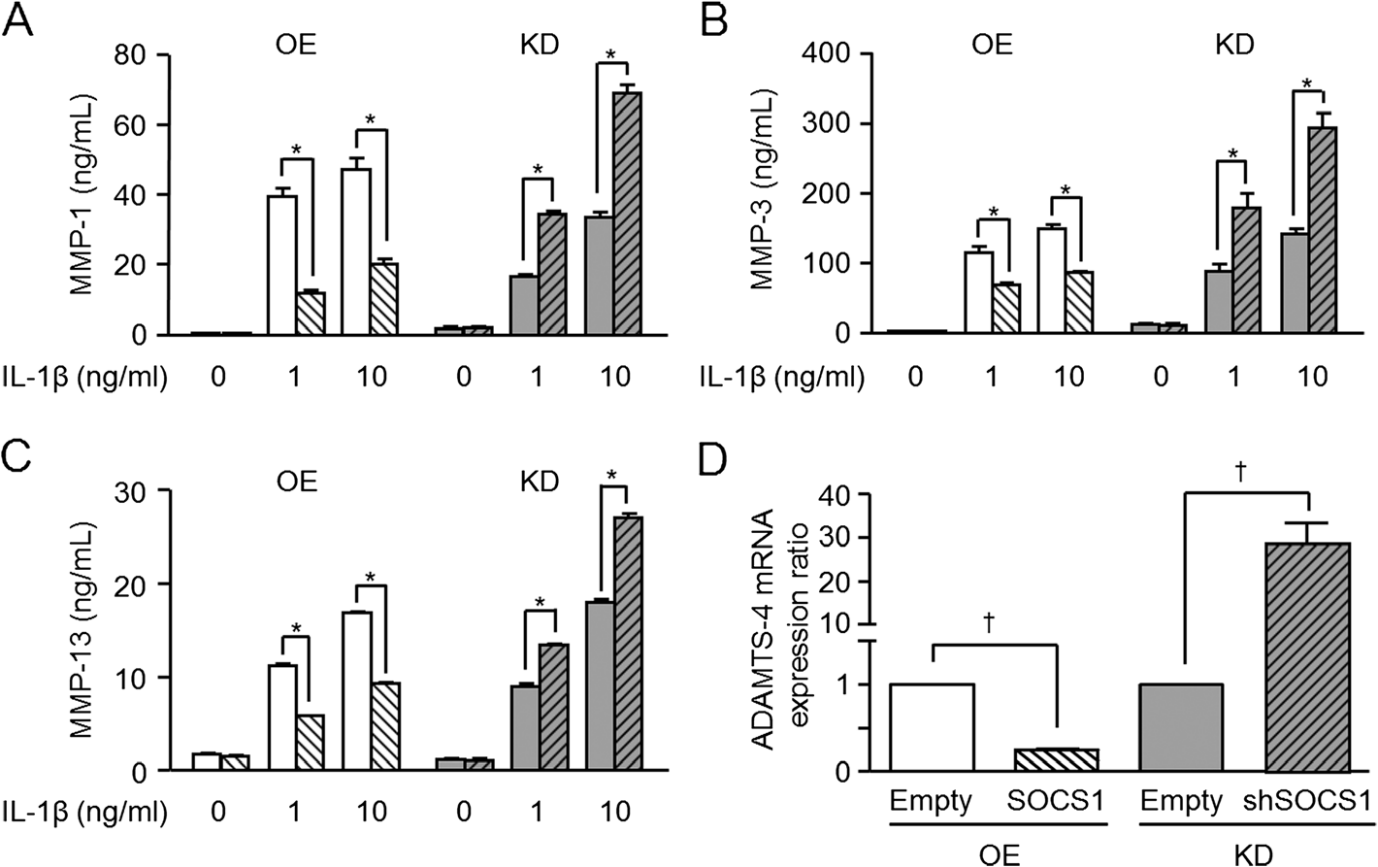
**SOCS1 overexpression inhibits and its knockdown enhances MMPs and ADAMTS-4 production in SW1353 cells.** The effect of SOCS1 over- or underexpression on MMPs secretion by SW1353 cells after IL-1β stimulation was investigated. After 24 hours, SOCS1-overexpressing SW1353 cells (white hatched bar) produced significantly lower amounts of MMP-1 **(A)**, MMP-3 **(B)**, and MMP-13 **(C)** than control SW1353 cells (white bar). Conversely, SOCS1-knockdown SW1353 cells (gray hatched bar) produced significantly higher amounts of MMP-1 **(A)**, MMP-3 **(B)**, and MMP-13 **(C)** than controls (gray bar). SOCS1 overexpression and knockdown showed a similar effect of SOCS1 on ADAMTS-4 transcript expression after IL-1β stimulation (10 ng/ml, **D**). Data were expressed as the mean ± SEM (*n* = 3). OE, overexpression; KD, knockdown; *P ≤ 0.005; †*P* < 0.05 by Mann–Whitney test.

Because MMPs production is induced by IL-1β, we evaluated the inhibitory effects of SOCS1 on MMPs synthesis by altering SOCS1 expression levels in the SW1353 chondrosarcoma cells. When nontransfected SW1353 cells were stimulated with IL-1β, MMP-1, MMP-3, and MMP-13 secretion were significantly increased (data not shown), consistent with previous reports [21]. In contrast, the SOCS1-overexpressing chondrocytes produced significantly lower levels of MMPs on addition of IL-1β (Figure 3A-C). Conversely, levels of MMP-1, MMP-3, and MMP-13 were significantly increased in the SOCS1-knockdown SW1353 cell line that was transfected with lentiviral SOCS1 shRNA (Figure 3A-C). The secretion of TIMP-1 from SOCS1-overexpressing or -knockdown cell lines was not altered under all of these conditions (data not shown). Also, ADAMTS-4 mRNA expression was suppressed in the SOCS1-overexpressing SW1353 cells and increased in the SOCS1-knockdown SW1353 cells (Figure 3D). These data suggest that SOCS1 effectively modulates the catabolic response of chondrocytes to IL-1β.

**Figure 4:**
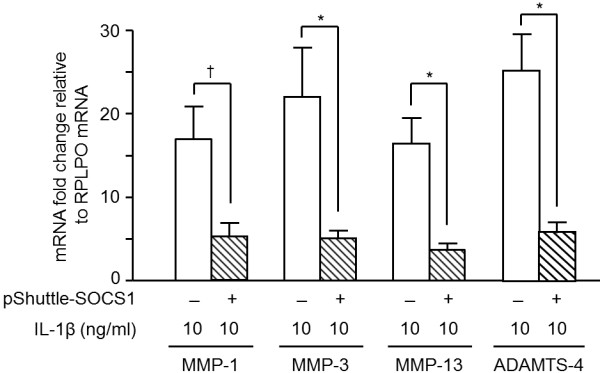
**Effects of SOCS1 overexpression on MMPs and ADAMTS4 expression in primary human articular chondrocytes (HACs).** HACs were electrotransfected with pShuttle-SOCS1 or empty vectors to overexpress SOCS1, as described in Methods. In the SOCS1-overexpressing HACs, IL-1β (10 ng/ml) induced MMPs and ADAMTS4 mRNA expression (white bars). Their expression was significantly lower by SOCS1 over-expressing (hatched bars). Data were expressed as the mean ± SEM (*n* = 4). *P < 0.005; †*P* < 0.05 by Wilcoxon signed-rank test.

To verify the inhibitory effects of SOCS1 in primary HACs, we investigated the changes in MMPs and ADAMTS-4 expression after IL-1β stimulation in HACs that were transiently electrotransfected with pShuttle2-SOCS1 vectors (*n* = 4). SOCS1 was increased at least by 19-fold compared with empty vector-transfected HACs. The IL-1β-induced MMPs and ADAMTS-4 mRNA expression levels were significantly downregulated in SOCS1 overexpressing HACs (*n* = 4; *P* < 0.05; Figure 4), similar to the SOCS1-overexpressing SW1353 cells.

### Effects of SOCS1 on MAPK and NF-κB signaling pathway

**Figure 5:**
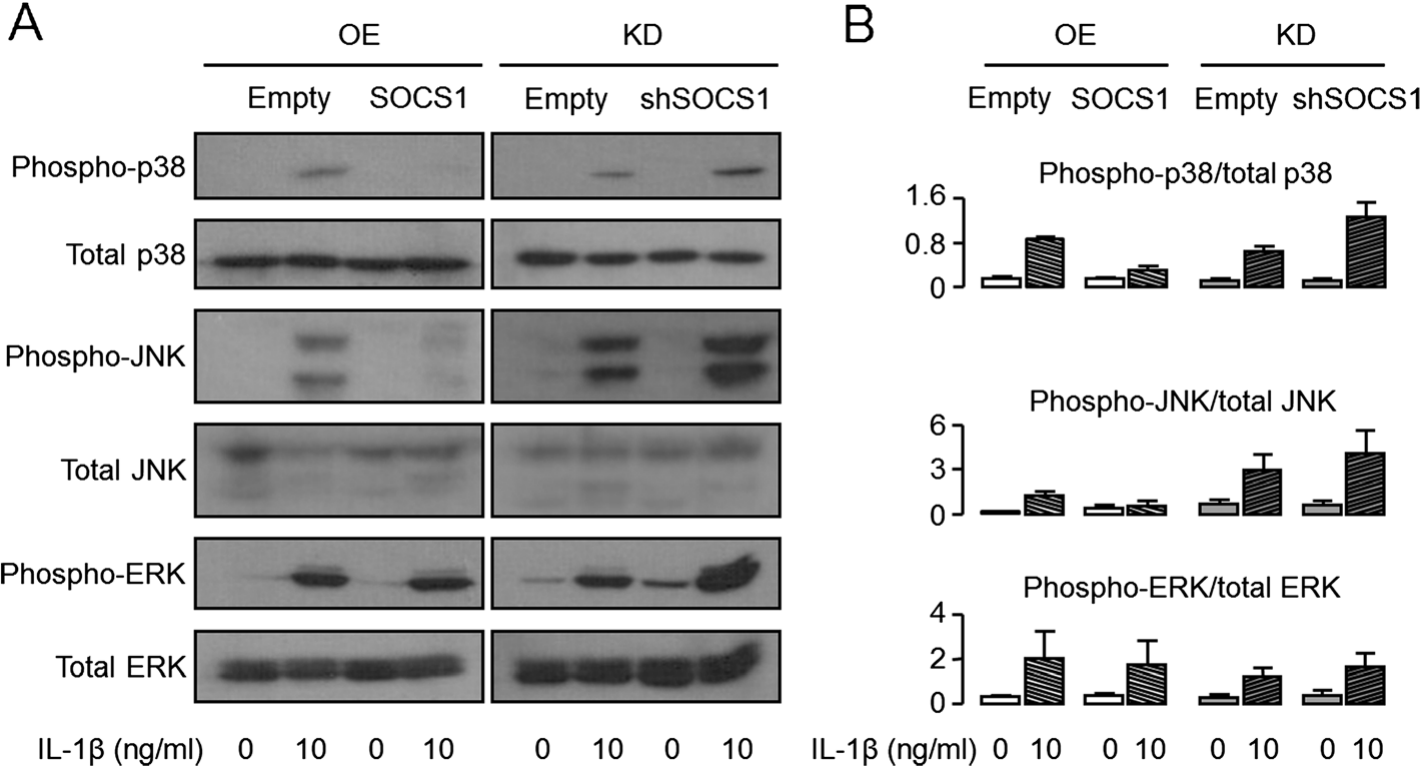
**Effects of SOCS1 on MAP kinase signaling.** SW1353 cells were transfected with SOCS1 or shSOCS1 vectors to overexpress or inhibit SOCS1, as described in Methods. A representative immunoblot image showed that SOCS1 overexpression decreased phosphorylation of p38 and JNK, whereas SOCS1 knockdown increased their phosphorylation in the presence of IL-1β (10 ng/ml, **A)**. The relative proportions of phosphorylated to total protein were determined with densitometry by using Image J software (version 1.48c, [22]; **B)**. Data were expressed as the means ± SEM (*n* = 3). OE, overexpression; KD, knockdown.

IL-1β signaling involves activation of both MAPK and NF-κB pathways. Indeed, SOCS1 overexpression decreased the phosphorylation level of p38 and JNK after IL-1β stimulation, whereas SOCS1 knockdown increased their phosphorylation (Figure 5).

**Figure 6:**
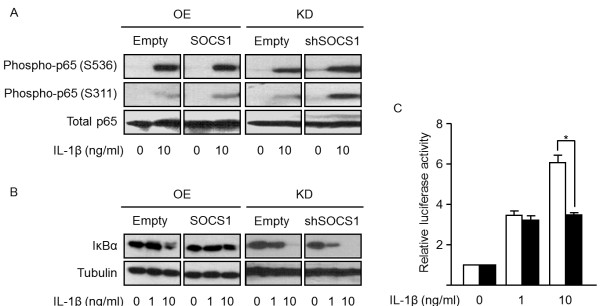
**Effects of SOCS1 on NF-κB signaling.** NF-κB p65 (Ser311 and Ser536) showed no alteration in phosphorylation after treatment with IL-1β (10 ng/ml) in SOCS1-overexpressing SW1353 cells, whereas SOCS1-knockdown cells increased levels of p65 phosphorylation at Ser311 **(A)**. The level of IκB was increased by SOCS1 overexpression and decreased by SOCS1 knockdown **(B)**. Accordingly, NF-κB-mediated luciferase activity was significantly reduced by SOCS1 overexpression after IL-1β treatment **(C)**. Data were expressed as the mean ± SEM (*n* = 3). OE, overexpression; KD, knockdown; *P < 0.005 by Mann–Whitney test.

After IL-1β stimulation, the phosphorylation levels of NF-κB p65 did not change at the serine 311 or 536 sites in the SOCS1-overexpressing cells, although the levels of phospho-NF-κB p65 (Ser311) were increased in the SOCS1-knockdown cells (Figure 6A). As NF-κB activity is controlled by the inhibitor protein IκB [23], we investigated the change in the amount of IκB. The SOCS1 overexpression prevented the IκB degradation, whereas the SOCS1 knockdown could not (Figure 6B). Accordingly, the NF-κB-dependent gene expression was significantly decreased in the SOCS1-overexpressing chondrocytes, as reflected by the low luciferase activity (*P* < 0.005; Figure 6C). These data suggest that SOCS1 inhibits NF-κB activity via preventing IκB from degradation.

**Figure 7:**
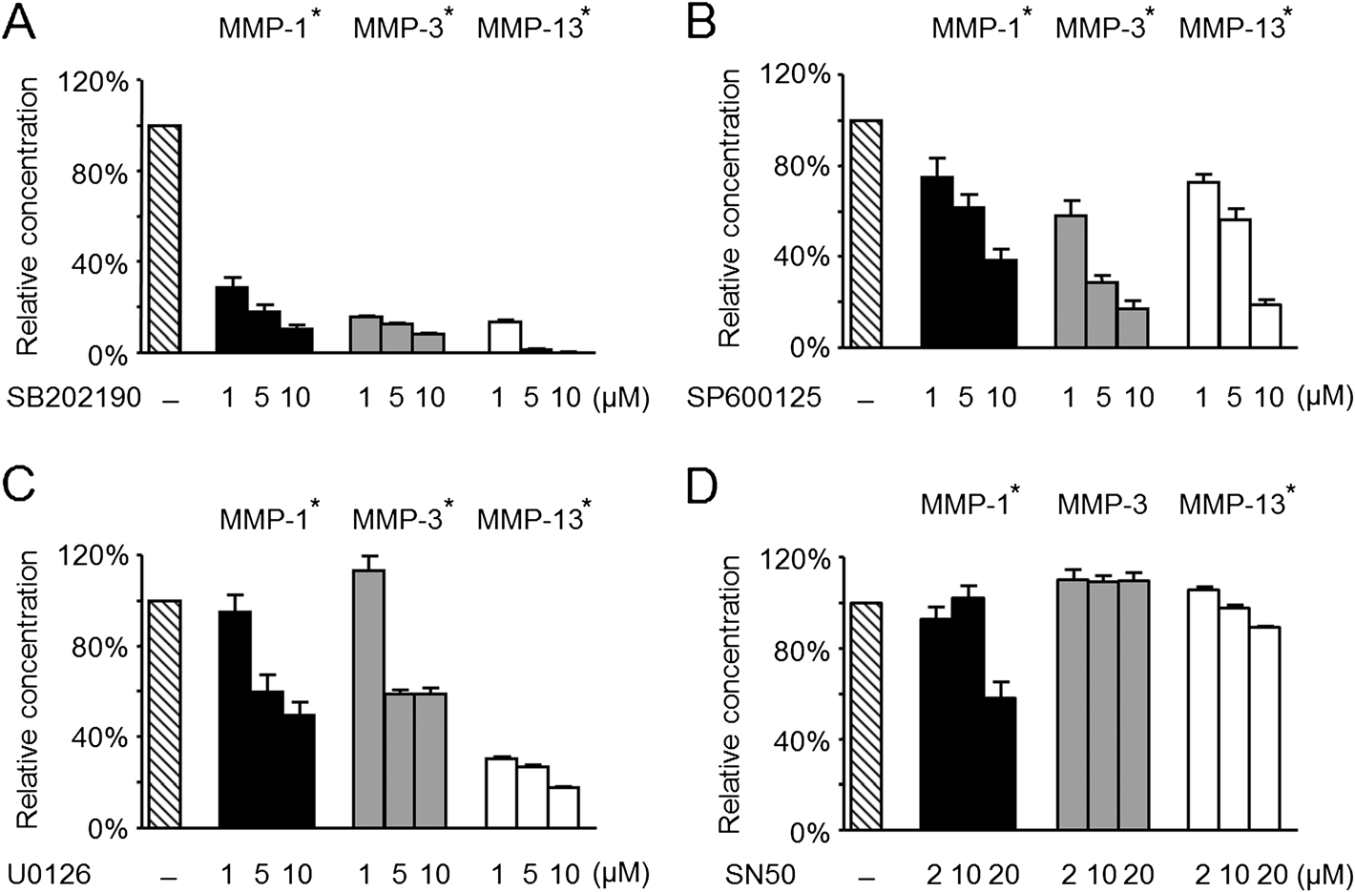
**Effect of MAP kinase and NF-κB inhibitors on MMPs secretion from SOCS1-knockdown SW1353 cells.** SOCS1-knockdown SW1353 cells were pretreated with inhibitors for 1 hour before stimulation with 10 ng/ml of IL-1β. After 24 hours, the levels of MMP-1, -3, and -13 were dramatically decreased by SB202190, a p38 MAP kinase inhibitor **(A)**. Blockade of C-JNK **(B)** and ERK **(C)** dose-dependently suppressed the secretion of MMPs from shSOCS1-transfected SW1353 cells. The production of MMP-1 and MMP-13 was partially inhibited with the SN50, a specific NF-κB inhibitory peptide **(D)**. Data were expressed as the mean ± SEM of relative MMPs levels, as compared with the control without inhibitors (*n* = 3). *P < 0.05 by Kruskal-Wallis test.

To ascertain the contributions of MAP kinase and NF-κB pathways to each MMP production, the SOCS1-knockdown chondrocytes were pretreated with various kinase inhibitors 1 hour before IL-1β stimulation. The p38 inhibitor SB202190 significantly suppressed the production of MMPs, even at a lower dose (*n* = 3, all *P* < 0.05; Figure 7A). JNK (SP600125) and ERK (U0126) inhibitors also inhibited MMPs secretion in a dose-dependent manner (all *P* < 0.05; Figure 7B and C). Although the effect of SN50 was less dramatic than that of MAP kinase inhibitors, blocking of NF-κB translocation reduced MMP-1 and MMP-13 production (Figure 7D).

### Effects of SOCS1 on TAK1 kinase

**Figure 8:**
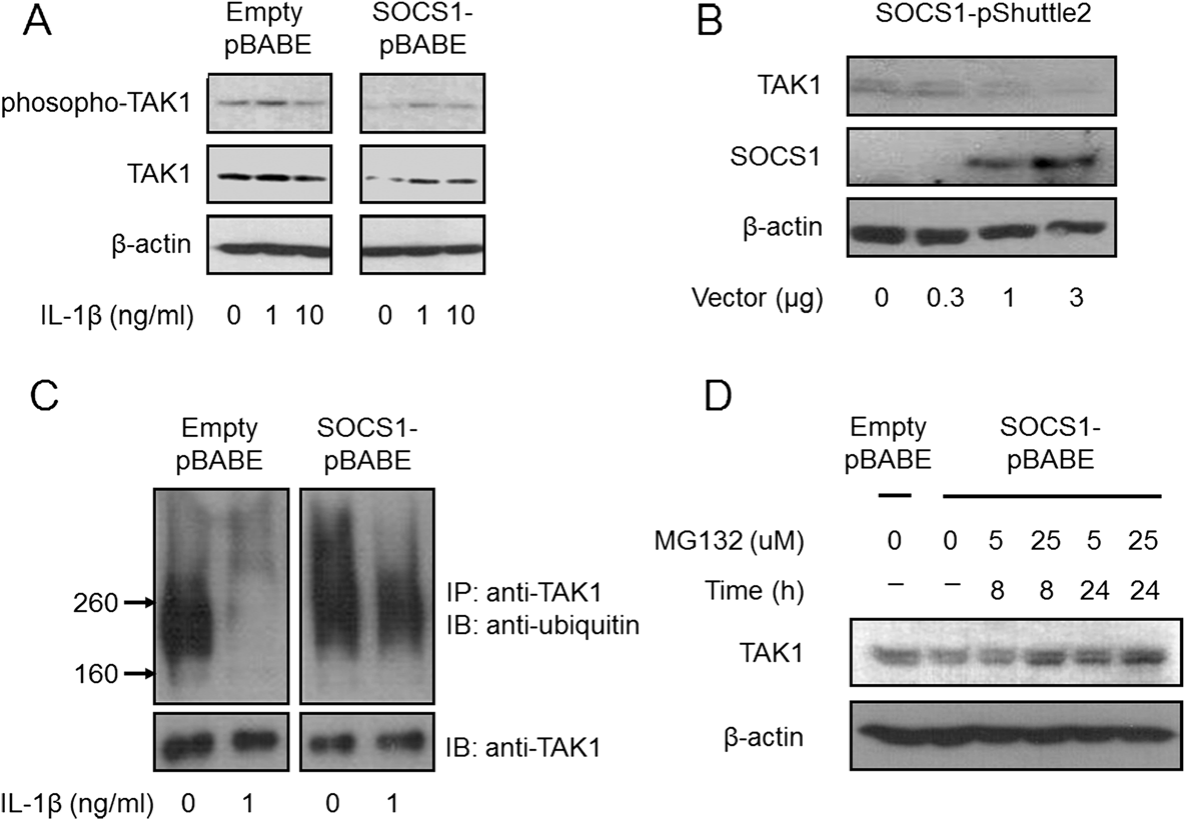
**Degradation of TAK1 via ubiquitination by SOCS1.** Representative immunoblots with phosphor-specific antibodies for TAK1 showed no alteration in phosphorylation after treatment with IL-1β in SOCS1-overexpressing SW1353 cells. However, TAK1 expression was reduced in pBABE-SOCS1-transfected SW1353 cells when compared with pBABE-transfected cells **(A)**. Twenty-four hours after transfection with increasing amounts of SOCS1, new protein synthesis was inhibited with 20 μg/ml cycloheximide for 8 hours. TAK1 levels decreased with transient SOCS1 transfection in a dose-dependent manner **(B)**. The level of TAK1 ubiquitination was analyzed via immunoprecipitation with anti-TAK1 and subsequent immunoblotting with anti-ubiquitin antibodies. The amount of ubiquitinated TAK1 protein was higher by SOCS1 overexpression **(C)**. The treatment of MG132 as a proteasome inhibitor dose- and time-dependently increased the levels of TAK1 protein in SOCS1-overexpressing SW1353 cells **(D)**. IP, immunoprecipitation; IB, immunoblotting.

TAK1 is a MAPK kinase kinase family protein that is activated by several cytokines, including IL-1β, and it is essential for MAPK and NF-κB activation. Frobøse *et al*. [24] reported that SOCS-3 inhibited the IL-1β-induced activity of TAK-1 in INS-1 cells, a rat pancreatic β-cell line [23]. Furthermore, SOCS1 was able to inhibit both MAPK and NF-κB signaling pathways in our models. Thus, we examined the effects of SOCS1 on TAK1 activity. Stable SOCS1 overexpression did not alter TAK1 phosphorylation levels after IL-1β treatment (Figure 8A). Unexpectedly, however, the levels of total TAK1 decreased in the SOCS1-overexpressing cells in a “gene dose”-dependent manner (Figure 8B). Because SOCS1 degrades intracellular proteins via ubiquitination, the ubiquitination level of TAK1 was investigated. Lysates of the SOCS1-overexpressing cells were immunoprecipitated by using anti-TAK1 antibodies. The SOCS1-overexpression led to a higher level of TAK1 ubiquitination after IL-1β stimulation (Figure 8C), suggesting TAK1 ubiquitination as a mechanism by which SOCS1 decreases the TAK1 levels. Additionally, when the SOCS1-overexpressing SW1353 cells were exposed to MG132, a proteasome inhibitor, TAK1 levels were increased in a time- and concentration-dependent manner (Figure 8D).

## Discussion

Cartilage damage in OA has been considered a result of an imbalance between catabolic and anabolic processes. A large body of the evidence reveals that proinflammatory cytokines are present in the synovial membrane and cartilage, even in the early stage of OA, and they function as major mediators of cartilage destruction [2,3]. IL-1β is believed to play a vital role as a major catabolic factor in OA cartilage. However, anti-IL-1β therapy, such as anakinra, did not provide any significant clinical benefit in OA patients [25,26]. Furthermore, paradoxically, the IL-1β-deficient mice accelerated a posttraumatic or spontaneous OA, and the IL-6-deficient male mice developed spontaneous knee OA [5-7]. These findings suggest that IL-1β and IL-6 paradoxically have a joint-protective role by a secondary regulatory system that counteracts the catabolic effects of inflammation. One such candidate is SOCS, which inhibits cellular inflammatory response as “a cytokine-inducible negative regulator of cytokine signaling” [8,9]. Interestingly, concerning the gender effect in IL-6-deficient mice, it was reported that estrogen or progesterone could increase the expression levels of SOCS1 [27,28]. Indeed, expression of SOCS1 was increased in OA cartilage in parallel to damage severity, and SOCS1 expression was directly induced by IL-1β in human articular chondrocytes in our study. Our experiments clearly showed suppressive effects of SOCS1 on IL-1β-induced MMPs and ADAMTS-4 production in human chondrocytes in both SOCS1-overexpression and -knockdown systems. These findings suggest that IL-1β-inducible SOCS1 acts as a negative regulator of IL-1β in human chondrocytes in OA pathogenesis, and the absent efficacy of anti-IL-1β treatment could, in part, result from the loss of this chondroprotective role of SOCS1. In addition, Fan *et al*. [29,30] reported that OA chondrocytes were less responsive to IL-1β than were normal chondrocytes. This could be explained by our observations; the higher SOCS1 expression in OA cartilage. However, as SOCS1-expressing chondrocytes were observed mainly in the area of severely damaged cartilage, and SOCS1 induction was only modest by IL-1β alone, the chondroprotective role of SOCS1 would be modest in areas of mild or moderate damage. Thus, in early OA, catabolic effects of IL-1β on cartilage overweigh the chondroprotection by inducible SOCS1. Further study is needed to address the possibility of SOCS1 as a novel therapeutic target for human OA.

To date, studies on the expression of the SOCS family have yielded inconsistent results in OA cartilage or chondrocytes. de Andrés *et al*. [31] reported that the SOCS1 and SOCS3 mRNA levels were similar in OA and normal chondrocytes, whereas SOCS2 and CIS-1 mRNA levels were suppressed in OA chondrocytes. Recently, van de Loo *et al*. [32] showed that the levels of SOCS1 mRNA expression in OA cartilage were comparable to those in normal cartilage, whereas SOCS3 mRNA and protein levels were significantly upregulated in OA cartilage. However, we demonstrated for the first time that SOCS1 protein is present in human cartilage, especially in the area of severe cartilage damage. The discrepancies between the findings may result from the different specimens, isolated chondrocytes versus cartilage tissue, and the different detection methods, that is, quantitative PCR versus IHC. Additionally, SOCS1 mRNA levels may be affected by passage numbers or culture methods. Nonetheless, our data confirm the inducibility of SOCS1 by IL-1β, consistent with the observation by van de Loo *et al*. They demonstrated a time-dependent increase in SOCS1 mRNA levels when OA chondrocytes were stimulated with 10 ng/ml of IL-1β or IFN-γ, with the increment in SOCS3 mRNA tending to decrease over time. Although SOCS3 was reported to reduce the anabolic action of insulin-like growth factor 1, SOCS3 overexpression in bovine chondrocytes decreased the production of IL-1β or lipopolysaccharide (LPS)-induced nitric oxide [14,32]. A recent study demonstrated that secreted factors from mesenchymal stem cells upregulated SOCS1 and decreased SOCS3 mRNA expression in OA cartilage [33].

In the present study, the inhibitory effects of SOCS1 on IL-1β actions were mediated by inhibition of p38 and JNK MAP kinases and NF-κB pathways. Since its initial discovery, SOCS1 has been known to exert a negative regulation on the JAK-STAT pathway [8,9]. But it was reported that overexpressed SOCS1 reduced p38, JNK, and ERK MAPK phosphorylation in adiponectin-stimulated RAW264 cells [34]. Additionally, it was observed that IFN-γ^(-/-)^SOCS1^(-/-)^ macrophages showed a great increment of LPS-induced p38 phosphorylation when compared with IFN-γ^(-/-)^SOCS1^(+/+)^ macrophages [35]. When taking into account the aforementioned data along with our results, the regulatory action of SOCS1 can apparently be mediated by inhibition of MAPK activation, apart from the JAK-STAT pathway.

Several reports have shown that SOCS1 is also able to regulate NF-κB signaling at different levels [16,36,37]. A group of German researchers [16,38] reported that SOCS1 has a nuclear localization signal and is predominantly localized in the nucleus, unlike CIS-1 and SOCS3. In the nucleus, NF-κB p65 bound to SOCS1 is degraded via ubiquitination with suppression of NF-κB-dependent gene expression. Indeed, in the present study, SCOS1 was present in the nucleus as well as in the cytoplasm of chondrocytes. In addition, NF-κB luciferase activity levels were reduced in the SOCS1-overexpressing cells in the presence of IL-1β. In this context, the inhibitory effects of SOCS1 on the IL-1β-induced MMP production may be partially mediated by degradation of p65. However, p65 or phosphor-p65 levels did not change with SOCS1 overexpression. Instead, the degradation of inhibitory IκB was suppressed in the SOCS1-overexpressing chondrocytes after stimulation with IL-1β. These findings are in line with previous findings that LPS-induced IκB degradation was delayed in the SOCS1-transfected RAW264 cells [34,35]. However, as shown in Figure 7, the antagonistic effect of SOCS1 on IL-1β signaling might not necessarily depend on the downregulation of the NF-κB pathway in human chondrocytes.

SOCS1 operated in both MAPK and NF-κB pathways in our study. TAK1 is a kinase that activates both IκB kinase and MAPK kinases (MKKs), and its activation leads to phosphorylation of p38, JNK, and ERK kinases and IκB degradation. Frobøse *et al*. [24] found that SOSC3 inhibited IL-1β signal transduction via suppression of the TRAF6 ubiquitination that is required for TAK1 activation. However, we did not observe any change in phosphorylation levels of TAK1 in the SOCS1-overexpressing cells. Rather, SOCS1 decreased the levels of TAK1 protein. The dose-dependent suppression of TAK1 protein was additionally confirmed by using a transient SOCS1-overexpression system. The SOCS box is a C-terminal domain of SOCS family proteins, including SOCS1, and it is essential to recruit the ubiquitin-transferase system. The domain can function as E3 ubiquitin ligases and mediate the ubiquitination and subsequent degradation of target proteins [9]. Thus, we examined the amount of ubiquitinated TAK1 in the SOCS1-overexpressing chondrocytes and found that ubiquitinated forms of TAK1 were easily detectable after IL-1β stimulation. Moreover, MG132 proteasome inhibitor increased TAK1 levels in SOCS1-overexpressing chondrocytes. These findings suggested that SOCS1 provides a novel negative-feedback mechanism through the degradation of TAK1, which is involved in IL-1β signaling [39].

Although the present study is the first to describe a novel role of SOCS1 in OA pathogenesis, this study has several limitations. First, we used an SOCS1 overexpression and knockdown system. Although the SOCS1 expression is increased in OA chondrocytes *in vivo*, the SOCS1 *in vitro* transfection could be overexpressed in supraphysiologic concentrations.

Second, our findings are limited to SOCS1 in chondrocytes, and they cannot reflect the real OA conditions in which many cell types are involved. Nonetheless, chondrocytes are considered critical to the OA process [40]. Because SOCS1 deficiency results in 100% perinatal lethality due to multiorgan inflammatory lesions [41], joint tissue-specific deletion approaches will probably be essential to further investigation of the role of SOCS1 on OA pathogenesis *in vivo*.

Third, we investigated the effect of SOCS1 on signaling pathways in chondrosarcoma SW1353 cell lines, not in primary human chondrocytes. However, SW1353 cells have been used as a well-established chondrocyte model in which the catabolic response after IL-1β treatment is similar to that in primary human articular chondrocytes [21].

## Conclusions

The IL-1β-inducible SOCS1 might mediate a joint-protective role in OA cartilage by inhibiting IL-1β signaling at multiple levels and by reducing levels of catabolic enzymes. Induction of SOCS1 might offer new therapeutic opportunities in OA treatment.

## Abbreviations

ADAMTS-4: Aggrecanase-1, a disintegrin and metalloproteinase with thrombospondin motifs 4; CIS-1: Cytokine-inducible SH2-containing protein 1; DMEM: Dulbecco modified Eagle medium; ELISA: Enzyme-linked immunosorbent assay; ERK: Extracellular-signal-regulated kinase; FBS: Fetal bovine serum; GAPDH: Glyceraldehyde-3-phosphate dehydrogenase; HAC: Human articular chondrocyte; IFN: Interferon; IHC: Immunohistochemistry; IP: Immunoprecipitation; IκB: Inhibitor of nuclear factor κB; JAK: Janus kinase; JNK: c-Jun N-terminal kinase; MAPK: Mitogen-activated protein kinase; MMP: Matrix metalloproteinase; NF-κB: Nuclear factor κB; OA: Osteoarthritis; OARSI: OsteoArthritis Research Society International; RT-PCR: Reverse transcription polymerase chain reaction; shRNA: Small hairpin RNA; SOCS: Suppressor of cytokine signaling; STAT: Signal transducer and activator of transcription; TAK1: Transforming growth factor-β activated kinase 1; TIMP: Tissue inhibitor of metalloproteinase; TNF: Tumor necrosis factor.

## Competing interests

The authors declare that they have no competing interests.

## Authors’ contributions

YSC and YJL conceived of and designed the study and were involved in the acquisition and interpretation of data. YSC and JKP prepared the initial draft of the manuscript. JKP, EHK, JMZ, WEC, and YWS participated in the analysis and interpretation of data and revised the manuscript. YKL and TKK participated in the design of the study, collected the OA cartilage samples, and were involved in the critical revision of the manuscript. JHC was involved in the acquisition and interpretation of immunohistochemical data and in the revision of the manuscript. All authors read and approved the final manuscript.

## References

[B1] Goldring MB Goldring SR Articular cartilage and subchondral bone in the pathogenesis of osteoarthritis Ann N Y Acad Sci 2010 15 230 237 10.1111/j.1749-6632.2009.05240.x 20392241

[B2] Loeser RF Molecular mechanisms of cartilage destruction: mechanics, inflammatory mediators, and aging collide Arthritis Rheum 2006 15 1357 1360 10.1002/art.21813 16645963 PMC1774815

[B3] Kapoor M Martel-Pelletier J Lajeunesse D Pelletier JP Fahmi H Role of proinflammatory cytokines in the pathophysiology of osteoarthritis Nat Rev Rheumatol 2011 15 33 42 10.1038/nrrheum.2010.196 21119608

[B4] Daheshia M Yao JQ The interleukin 1beta pathway in the pathogenesis of osteoarthritis J Rheumatol 2008 15 2306 2312 10.3899/jrheum.080346 18925684

[B5] Clements KM Price JS Chambers MG Visco DM Poole AR Mason RM Gene deletion of either interleukin-1beta, interleukin-1beta-converting enzyme, inducible nitric oxide synthase, or stromelysin 1 accelerates the development of knee osteoarthritis in mice after surgical transection of the medial collateral ligament and partial medial meniscectomy Arthritis Rheum 2003 15 3452 3463 10.1002/art.11355 14673996

[B6] Blom AB Van Lent PL van der Kraan PM Joosten LA van den Berg WB Differential role for interleukin-1 in induced instability osteoarthritis and spontaneously occurring osteoarthritis in mice [abstract] Osteoarthritis Cartilage 2004 15 S90

[B7] de Hooge AS van de Loo FA Bennink MB Arntz OJ de Hooge P van den Berg WB Male IL-6 gene knock out mice developed more advanced osteoarthritis upon aging Osteoarthritis Cartilage 2005 15 66 73 10.1016/j.joca.2004.09.011 15639639

[B8] Davey GM Heath WR Starr R SOCS1: a potent and multifaceted regulator of cytokines and cell-mediated inflammation Tissue Antigens 2006 15 1 9 16451196 10.1111/j.1399-0039.2005.00532.x

[B9] Yoshimura A Naka T Kubo M SOCS proteins, cytokine signalling and immune regulation Nat Rev Immunol 2007 15 454 465 10.1038/nri2093 17525754

[B10] Diao Y Wang X Wu Z SOCS1, SOCS3, and PIAS1 promote myogenic differentiation by inhibiting the leukemia inhibitory factor-induced JAK1/STAT1/STAT3 pathway Mol Cell Biol 2009 15 5084 5093 10.1128/MCB.00267-09 19620279 PMC2738280

[B11] Song MM Shuai K The suppressor of cytokine signaling (SOCS) 1 and SOCS3 but not SOCS2 proteins inhibit interferon-mediated antiviral and antiproliferative activities J Biol Chem 1998 15 35056 35062 10.1074/jbc.273.52.35056 9857039

[B12] Wong PK Egan PJ Croker BA O'Donnell K Sims NA Drake S Kiu H McManus EJ Alexander WS Roberts AW Wicks IP SOCS-3 negatively regulates innate and adaptive immune mechanisms in acute IL-1-dependent inflammatory arthritis J Clin Invest 2006 15 1571 1581 10.1172/JCI25660 16710471 PMC1462939

[B13] Deon D Ahmed S Tai K Scaletta N Herrero C Lee IH Krause A Ivashkiv LB Cross-talk between IL-1 and IL-6 signaling pathways in rheumatoid arthritis synovial fibroblasts J Immunol 2001 15 5395 5403 11673558 10.4049/jimmunol.167.9.5395

[B14] Smeets RL Veenbergen S Arntz OJ Bennink MB Joosten LA van den Berg WB van de Loo FA A novel role for suppressor of cytokine signaling 3 in cartilage destruction via induction of chondrocyte desensitization toward insulin-like growth factor Arthritis Rheum 2006 15 1518 1528 10.1002/art.21752 16646036

[B15] Chong MM Metcalf D Jamieson E Alexander WS Kay TW Suppressor of cytokine signaling-1 in T cells and macrophages is critical for preventing lethal inflammation Blood 2005 15 1668 1675 10.1182/blood-2004-08-3049 15899915

[B16] Strebovsky J Walker P Lang R Dalpke AH Suppressor of cytokine signaling 1 (SOCS1) limits NFkappaB signaling by decreasing p65 stability within the cell nucleus FASEB J 2011 15 863 874 10.1096/fj.10-170597 21084693

[B17] Mitchell T Sugden B Stimulation of NF-kappa B-mediated transcription by mutant derivatives of the latent membrane protein of Epstein-Barr virus J Virol 1995 15 2968 2976 7707523 10.1128/jvi.69.5.2968-2976.1995 PMC188996

[B18] Kang EH Lee YJ Kim TK Chang CB Chung JH Shin K Lee EY Lee EB Song YW Adiponectin is a potential catabolic mediator in osteoarthritis cartilage Arthritis Res Ther 2010 15 R231 10.1186/ar3218 21194467 PMC3046544

[B19] Pritzker KP Gay S Jimenez SA Ostergaard K Pelletier JP Revell PA Salter D van den Berg WB Osteoarthritis cartilage histopathology: grading and staging Osteoarthritis Cartilage 2006 15 13 29 10.1016/j.joca.2005.07.014 16242352

[B20] Koelsche C Strebovsky J Baetz A Dalpke AH Structural and functional analysis of a nuclear localization signal in SOCS1 Mol Immunol 2009 15 2474 2480 10.1016/j.molimm.2009.05.020 19515423

[B21] Gebauer M Saas J Sohler F Haag J Söder S Pieper M Bartnik E Beninga J Zimmer R Aigner T Comparison of the chondrosarcoma cell line SW1353 with primary human adult articular chondrocytes with regard to their gene expression profile and reactivity to IL-1beta Osteoarthritis Cartilage 2005 15 697 708 10.1016/j.joca.2005.04.004 15950496

[B22] http://rsbweb.nih.gov/ij/

[B23] Hoffmann A Levchenko A Scott ML Baltimore D The IkappaB-NF-kappaB signaling module: temporal control and selective gene activation Science 2002 15 1241 1245 10.1126/science.1071914 12424381

[B24] Frobøse H Rønn SG Heding PE Mendoza H Cohen P Mandrup-Poulsen T Billestrup N Suppressor of cytokine signaling-3 inhibits interleukin-1 signaling by targeting the TRAF-6/TAK1 complex Mol Endocrinol 2006 15 1587 1596 16543409 10.1210/me.2005-0301

[B25] Chevalier X Goupille P Beaulieu AD Burch FX Bensen WG Conrozier T Loeuille D Kivitz AJ Silver D Appleton BE Intraarticular injection of anakinra in osteoarthritis of the knee: a multicenter, randomized, double-blind, placebo-controlled study Arthritis Rheum 2009 15 344 352 10.1002/art.24096 19248129

[B26] Cohen SB Proudman S Kivitz AJ Burch FX Donohue JP Burstein D Sun YN Banfield C Vincent MS Ni L Zack DJ A randomized, double-blind study of AMG 108 (a fully human monoclonal antibody to IL-1R1) in patients with osteoarthritis of the knee Arthritis Res Ther 2011 15 R125 10.1186/ar3430 21801403 PMC3239365

[B27] Steyn FJ Anderson GM Grattan DR Hormonal regulation of suppressors of cytokine signaling (SOCS) messenger ribonucleic acid in the arcuate nucleus during late pregnancy Endocrinology 2008 15 3206 3214 10.1210/en.2007-1623 18325991

[B28] Su L Sun Y Ma F Lü P Huang H Zhou J Progesterone inhibits Toll-like receptor 4-mediated innate immune response in macrophages by suppressing NF-kappaB activation and enhancing SOCS1 expression Immunol Lett 2009 15 151 155 10.1016/j.imlet.2009.07.003 19607861

[B29] Fan Z Bau B Yang H Aigner T IL-1beta induction of IL-6 and LIF in normal articular human chondrocytes involves the ERK, p38 and NFkappaB signaling pathways Cytokine 2004 15 17 24 10.1016/j.cyto.2004.06.003 15341921

[B30] Fan Z Bau B Yang H Soeder S Aigner T Freshly isolated osteoarthritic chondrocytes are catabolically more active than normal chondrocytes, but less responsive to catabolic stimulation with interleukin-1beta Arthritis Rheum 2005 15 136 143 10.1002/art.20725 15641077

[B31] de Andrés MC Imagawa K Hashimoto K Gonzalez A Goldring MB Roach HI Oreffo RO Suppressors of cytokine signalling (SOCS) are reduced in osteoarthritis Biochem Biophys Res Commun 2011 15 54 59 10.1016/j.bbrc.2011.02.101 21352802 PMC3937865

[B32] van de Loo FA Veenbergen S van den Brand B Bennink MB Blaney-Davidson E Arntz OJ van Beuningen HM van der Kraan PM van den Berg WB Enhanced suppressor of cytokine signaling (SOCS)-3 in arthritic cartilage dysregulates human chondrocyte function Arthritis Rheum 2012 15 3313 3323 10.1002/art.34529 22576756

[B33] van Buul GM Villafuertes E Bos PK Waarsing JH Kops N Narcisi R Weinans H Verhaar JA Bernsen MR van Osch GJ Mesenchymal stem cells secrete factors that inhibit inflammatory processes in short-term osteoarthritic synovium and cartilage explant culture Osteoarthritis Cartilage 2012 15 1186 1196 10.1016/j.joca.2012.06.003 22771777

[B34] Fujimoto A Akifusa S Hirofuji T Yamashita Y Involvement of suppressor of cytokine signaling-1 in globular adiponectin-induced granulocyte colony-stimulating factor in RAW 264 cell Mol Immunol 2011 15 2052 2058 10.1016/j.molimm.2011.06.440 21764457

[B35] Kinjyo I Hanada T Inagaki-Ohara K Mori H Aki D Ohishi M Yoshida H Kubo M Yoshimura A SOCS1/JAB is a negative regulator of LPS-induced macrophage activation Immunity 2002 15 583 591 10.1016/S1074-7613(02)00446-6 12433365

[B36] Ryo A Suizu F Yoshida Y Perrem K Liou YC Wulf G Rottapel R Yamaoka S Lu KP Regulation of NF-kappaB signaling by Pin1-dependent prolyl isomerization and ubiquitin-mediated proteolysis of p65/RelA Mol Cell 2003 15 1413 1426 10.1016/S1097-2765(03)00490-8 14690596

[B37] Mansell A Smith R Doyle SL Gray P Fenner JE Crack PJ Nicholson SE Hilton DJ O'Neill LA Hertzog PJ Suppressor of cytokine signaling 1 negatively regulates Toll-like receptor signaling by mediating Mal degradation Nat Immunol 2006 15 148 155 16415872 10.1038/ni1299

[B38] Baetz A Koelsche C Strebovsky J Heeg K Dalpke AH Identification of a nuclear localization signal in suppressor of cytokine signaling 1 FASEB J 2008 15 4296 4305 10.1096/fj.08-116079 18725457

[B39] Klatt AR Klinger G Neumüller O Eidenmüller B Wagner I Achenbach T Aigner T Bartnik E TAK1 downregulation reduces IL-1beta induced expression of MMP13, MMP1 and TNF-alpha Biomed Pharmacother 2006 15 55 61 10.1016/j.biopha.2005.08.007 16459052

[B40] Goldring MB The role of the chondrocyte in osteoarthritis Arthritis Rheum 2000 15 1916 1926 10.1002/1529-0131(200009)43:9<1916::AID-ANR2>3.0.CO;2-I 11014341

[B41] Marine JC Topham DJ McKay C Wang D Parganas E Stravopodis D Yoshimura A Ihle JN SOCS1 deficiency causes a lymphocyte-dependent perinatal lethality Cell 1999 15 609 616 10.1016/S0092-8674(00)80048-3 10490100

